# Reconstruction of Large Skeletal Defects: Current Clinical Therapeutic Strategies and Future Directions Using 3D Printing

**DOI:** 10.3389/fbioe.2020.00061

**Published:** 2020-02-12

**Authors:** Luciano Vidal, Carina Kampleitner, Meadhbh Á. Brennan, Alain Hoornaert, Pierre Layrolle

**Affiliations:** ^1^INSERM, UMR 1238, PHY-OS, Bone Sarcomas and Remodeling of Calcified Tissues, Faculty of Medicine, University of Nantes, Nantes, France; ^2^Department of Pharmacology and Toxicology, University of Vienna, Vienna, Austria; ^3^Harvard School of Engineering and Applied Sciences, Harvard University, Cambridge, MA, United States; ^4^CHU Nantes, Department of Implantology, Faculty of Dental Surgery, University of Nantes, Nantes, France

**Keywords:** large bone defects, bone regeneration, tissue engineering, vascularization, three-dimensional printing

## Abstract

The healing of bone fractures is a well-orchestrated physiological process involving multiple cell types and signaling molecules interacting at the fracture site to replace and repair bone tissue without scar formation. However, when the lesion is too large, normal healing is compromised. These so-called non-union bone fractures, mostly arising due to trauma, tumor resection or disease, represent a major therapeutic challenge for orthopedic and reconstructive surgeons. In this review, we firstly present the current commonly employed surgical strategies comprising auto-, allo-, and xenograft transplantations, as well as synthetic biomaterials. Further to this, we discuss the multiple factors influencing the effectiveness of the reconstructive therapy. One essential parameter is adequate vascularization that ensures the vitality of the bone grafts thereby supporting the regeneration process, however deficient vascularization presents a frequently encountered problem in current management strategies. To address this challenge, vascularized bone grafts, including free or pedicled fibula flaps, or *in situ* approaches using the Masquelet induced membrane, or the patient’s body as a bioreactor, comprise feasible alternatives. Finally, we highlight future directions and novel strategies such as 3D printing and bioprinting which could overcome some of the current challenges in the field of bone defect reconstruction, with the benefit of fabricating personalized and vascularized scaffolds.

## Introduction

The reconstruction of large bone defects caused by trauma, disease or tumor resection is a fundamental challenge for orthopedic and plastic surgeons. Their critical size exceeds the intrinsic capacity of self-regeneration and consequently bone repair is delayed and impaired. This type of lesion is termed non-union bone fracture and requires additional treatment with bone graft materials in order to restore pre-existing function (Dimitriou et al., 2011). Successful bone augmentation procedures should include an osteoconductive scaffold with sufficient mechanical stability, an osteoinductive stimulus to induce osteogenesis, and should enable osseointegration and vascularity (Albrektsson and Johansson, 2001; Giannoudis et al., 2008). The currently available treatment strategies of bone loss are based on autologous, allogeneic or xenogeneic bone transplantation, as well as synthetic biomaterials. Although autologous bone grafting still represents the gold standard technique for large bone reconstruction, several factors limit its application. A major restricting parameter is the volume of bone needed to treat this type of injury, as well as the associated pain and possible donor site complications due to the additional surgical intervention at the bone harvest site. Similar disadvantages may be observed for allogenic bone grafts including immunogenic reactions and transfer of diseases (Aro and Aho, 1993). Furthermore, many of these standard clinical grafting approaches fail due to the lack of adequate vascularization. Insufficient vascularity of the fracture site reduces the exchange of gas, nutrients and waste between the tissue and the blood system, as well as the delivery of cells to the site of injury, leading to inner graft necrosis (Mercado-Pagan et al., 2015; Fernandez de Grado et al., 2018). To circumvent this problem, vascularized bone transfers represent an excellent option that ensures bone vitality and avoids graft resorption. Nevertheless, complex fractures and their reconstructions require modeling of the transferred bone to adapt to the anatomical shape and extensive microsurgical techniques to connect the graft to the blood system. Some patient bioreactor attempts have also been made whereby a customized bone graft is implanted ectopically in the patient for several weeks before transferring it into the bone defect. Innovative fabrication approaches in the field of bone tissue engineering include three-dimensional (3D) printing and bioprinting to enable *ex vivo* personalized bone grafts based on anatomical medical imaging. They are generally composed of calcium phosphate/polymer composites or porous titanium. To enhance the material healing properties, 3D printed scaffolds can potentially include cells, growth factors, and vasculature. In this review, we present the current techniques clinically available for the reconstruction of critical-sized bone defects and point out future challenges and possibilities of new treatment modalities using customized and vascularized bone grafts with a focus on 3D printing and bioprinting fabrication methods.

## Present Management Strategies for Large Bone Lesions

**FIGURE 1:**
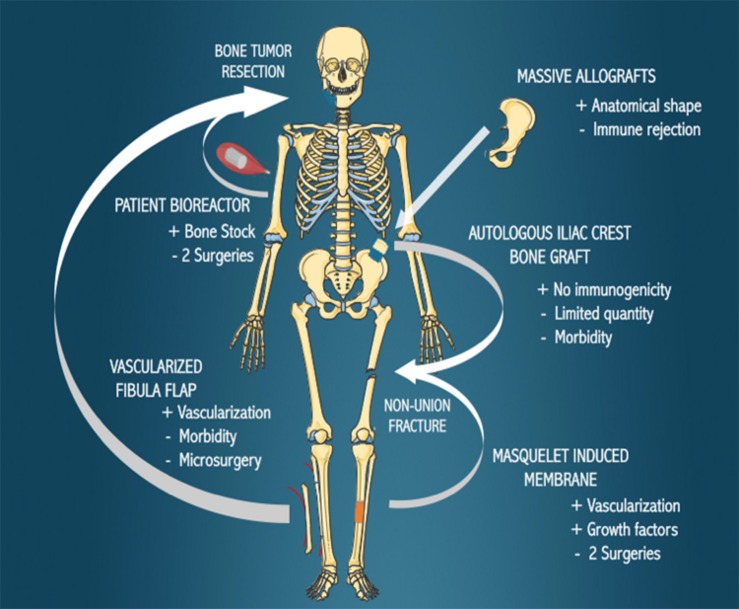
Current biological bone reconstruction techniques. Bone defects arising due to the resection of tumors or non-union fractures can be treated with the various methods indicated, with the benefits (+) and disadvantages (−) of each technique outlined.

The current reconstructive options for large bone defects, including autologous iliac grafting, autologous vascularized fibula transplantation, Masquelet’s induced membrane, massive allografts and *in vivo* patient bioreactor strategies are presented in Figure 1 and discussed in this section.

### Bone Grafts

The leading treatment for bone defect reconstruction remains bone grafting. The purpose of a bone graft is to support the repair process through osteoinduction, osteoconduction, and osteogenesis (Albrektsson and Johansson, 2001; Oryan et al., 2014). They can be categorized into different types based on the tissue source: autologous, allogeneic and xenogeneic bone grafts, as well as synthetic and biological biomaterials (Brydone et al., 2010). The selection of the ideal bone graft depends on several factors including the geometry, size and tissue viability of the bone defect, the biological and biomechanically characteristics of the bone graft, and the known advantages and associated complications of each graft option (Laurencin et al., 2014).

#### Autografts

**FIGURE 2:**
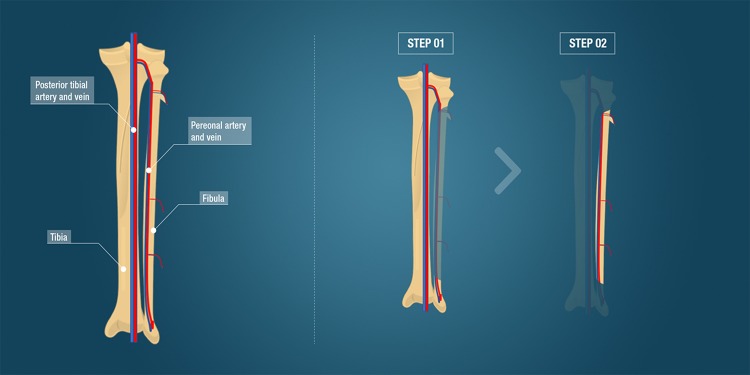
Fibula free vascularized flap. The anatomy including the tibia, fibula and major vessels is indicated. The surgical steps comprising the fibula flap, the gold standard clinical technique for large bone defect reconstruction, is demonstrated. Step 1 illustrates the flap dissection to obtain the bone flap with its vascular pedicle. Step 2 represents the bone flap with its vascular pedicle ready to be transplanted to the bone defect.

Autologous bone grafting, still the clinical standard reconstruction technique, entails harvesting bone tissue from an anatomical donor site and transplanting it to the recipient defect site (Sanan and Haines, 1997). The iliac crest is the preferred harvesting site for this type of transplant, whereby approximately 20 cm^3^ of cancellous bone is collected and used as a bone block or morselized into bone chips in order to fill a bone defect (Athanasiou et al., 2010). Autologous bone contains the patient’s own osteogenic cells and osteoinductive proteins, such as bone morphogenetic protein 2 (BMP2), BMP7, and platelet-derived growth factor (PDGF), providing optimal osteogenic, osteoinductive, and osteoconductive properties without risk of viral transmissions, while pain, hematoma, possible visceral injuries at the donor site and extended surgery time because of the two surgical sites are the main drawbacks (Albrektsson and Johansson, 2001; Parikh, 2002). Another disadvantage of cancellous bone grafting is that large amounts of bone graft cannot be obtained for critical-sized defect reconstruction (Oryan et al., 2013). Successful repair depends on osteogenic cell survival and tissue viability after transplantation to the recipient site, while neovascularization plays a determinant role. To overcome the disadvantage of limited vascularization, free vascularized bone flaps have been employed. Taylor et al. reported the first successful large bone defect reconstruction using a free vascularized bone transfer (Taylor et al., 1975). Vascularized bone grafts, such as an autologous vascularized fibula flap, iliac crest flap, rib flap, and radius flap, allow the reconstruction of large bone defects and are often used as a last resort to avoid limb amputation for patients. Fibula and iliac crest flaps have been used for the pelvis, head of long bones, and maxillofacial reconstruction. Free vascularized bone flaps are particularly suitable for mandible reconstructions after ballistic trauma or tumor resections. An optimal option for large bone defect reconstruction using autografts is a vascularized cortical autograft (Rizzo and Moran, 2008). Mandible reconstruction is predominantly performed by a fibula flap. Another option described in the literature for a hemimandible reconstruction is the iliac crest flap that has an adequate bone height to ensure osseointegration (Taylor, 1982, 1983, 1985) and allows optimal shape reconstruction of the mandible ramus. The fibula is dissected, harvested with a vascular pedicle, shaped and transplanted into the bone defect where it is reconnected to the local vasculature (Figure 2). This vascularized bone graft contains the patient’s own cells, growth factors and a vascularization bed thereby reducing graft resorption, enhancing healing and permitting better diffusion of antibiotics. Hidalgo et al. evaluated the fibula flap for mandible reconstruction and reported long-term outstanding functional and aesthetic results without bone resorption in non-irradiated and irradiated patients (Hidalgo and Pusic, 2002). Free fibula flap transfers for mandibular and maxillary reconstruction achieved 98.7% graft survival in some studies (Peng et al., 2005; Taylor et al., 2016). Further to this, pelvic ring reconstruction employing a double-barreled free vascularized autologous patient fibula graft after resection of malignant pelvic bone tumors was reported (Ogura et al., 2015). Additionally, lumbosacral spinal defects reconstruction was also achieved with the use of a fibula flap (Moran et al., 2009). The major complications of free vascularized bone flaps are post-operative vascular thrombosis and hence failure and free flap loss. The fibula flap requires laborious microsurgery to reconnect to the vasculature, and the need for sculpting of the graft to fit the anatomy of the bone defect. Furthermore, this technique requires extended anesthesia, specialized technical surgical skills and the sacrifice of blood vessels.

#### Allografts

Bone allografts are harvested from living donors during joint replacement (e.g., femoral heads) or from cadavers, and stored frozen and processed and transplanted into another patient (Keating and McQueen, 2001). Given the limitations of autografts, allografts became an alternative to large bone defect reconstruction. Allografts are used as powders, chips or complete bone structural forms, so called massive allografts and can be provided as a fresh graft, fresh-frozen, freeze-dried, demineralized, de-lipidized by solvents or supercritical carbon dioxide, and sterilized by irradiation (Bostrom and Seigerman, 2005; Zimmermann and Moghaddam, 2011). The primary advantage of allografts is their immediate availability in different sizes and shapes (Muscolo et al., 2004). They are composed of the extracellular bone matrix containing growth factors that stimulate regeneration, do not present complications associated with donor site harvesting, and present favorable mechanical strength (Mankin et al., 1996). For these reasons, allografts are particularly interesting for complex skeletal reconstruction after resection of bone tumors in pelvic bones of young patients. However, allografts present variable osteoinductive and osteoconductive properties and have lower osteogenic potential compared to autografts (Coquelin et al., 2012). Other disadvantages are the possibility of immune rejection and disease transmission (Aro and Aho, 1993). To overcome the latter disadvantage, Capanna et al. (1993) described a technique for the reconstruction of large metadiaphyseal bone defects, combining a massive allograft to support a centrally located autologous fibula flap with the aim of improving allograft incorporation and decreasing the risk of mechanical instability. This technique has proven efficacy for large bone defect reconstruction (Bakri et al., 2008). Other clinical studies described the use of allografts alone or associated with other therapies such as autologous concentrated bone marrow-derived cells (Putzier et al., 2009; Faldini et al., 2011; Scaglione et al., 2014).

#### Xenografts

Xenografts are harvested from different species and transplanted for patient bone defect repair, and the most commonly used are of bovine, porcine, or coral origin. The primary advantages are the high availability, favorable porosity for bone tissue ingrowth and comparable mechanical strength to native bone. However, similar to allografts, xenografts, when treated for clinical use, may lose part of their osteoinductive and osteoconductive abilities (Dimitriou et al., 2011). Moreover, a significant disadvantage of xenografts is the possible transmission of zoonotic diseases and immune rejection. Finally, xenografts have ethical and religious concerns. Karalashvili et al. (2017) described the use of a decellularized bovine bone graft in a zygomatic large bone defect reconstruction and reported long-term retention of graft shape without resorption and bone integration. Bovine cancellous xenografts have also been used in the treatment of tibial fractures in elderly patients and showed favorable healing outcomes (Bansal et al., 2009). However, the number of published studies using xenografts in large bone defect reconstruction is still limited and indeed clinical trials using bovine bone have shown poor results, describing graft rejection and failure in host tissue integration (Elliot and Richards, 2011; Patil et al., 2011; Shibuya et al., 2012; Ledford et al., 2013).

#### Synthetic Biomaterials

Langer and Vacanti described tissue engineering by the use of biocompatible materials associated with cells and/or biological factors, in order to replace or repair tissues or organs. Various biomaterials have been employed in the treatment of bone defects. Calcium phosphate ceramics (CaP ceramics) are synthetic materials composed of calcium hydroxyapatites (HA), therefore possessing a composition similar to the native bone matrix. CaP ceramics are primarily produced by sintering at high temperatures and are available with variable porosity and in construct or granules format, with their main advantage being their osteoconductivity (Albrektsson and Johansson, 2001; Lee et al., 2006; Samavedi et al., 2013). CaP ceramics most commonly employed in bone reconstruction are biphasic calcium phosphate (BCP), tricalcium phosphate (TCP), and HA. HA presents excellent osteoconductive and osseointegration properties and their macroporosity and pore interconnectivity allow excellent cell adhesion and proliferation, leading to osteoconduction and osteoinduction after transplantation *in vivo*, as well as revascularization of the implant (Bucholz et al., 1987; Eggli et al., 1988). TCP has higher pore interconnectivity than HA which is crucial for neovascularization and osteoconduction (Ogose et al., 2006), however, this higher interconnectivity gives TCP lower mechanical properties compared to HA and TCP is reabsorbed faster than HA after implantation (Torres et al., 2011). BCP is the combination of TCP and HA. BCP exploits the main advantages of both TCP and HA as they can be combined in various ratios (Daculsi et al., 1989). Calcium phosphate cement (CPC) differs from calcium phosphate ceramics because they are made at ambient temperatures from hydrolysis and are regarded as biomimetic. CPC can be used as filler by injection and for creating 3D printing constructs (Brown and Chow, 1983; Brown, 1987; Bertol et al., 2016), however, their slow degradation may delay bone formation (Lodoso-Torrecilla et al., 2018). Bioactive glass or bioglass is a synthetic silicate-based ceramic. It is rapidly resorbed in the first 2 weeks after implantation allowing a rapid new bone and vascularized implant ingrowth (Gerhardt and Boccaccini, 2010; Kurien et al., 2013). Synthetic bone substitutes are an excellent alternative to biological grafts in small bone defect reconstruction. However, due to the insufficient strength to sustain the body load and insufficient neovascularization ingrowth, bone substitutes are not the best option for large bone defect reconstruction (Stanovici et al., 2016). Their association with recombinant human growth factors and/or stem cell therapies could be a solution for this main disadvantage (Gomez-Barrena et al., 2011, 2019). Orthounion is an ongoing clinical trial studying the use of bone marrow mesenchymal stem cells combined with a bone substitute to fill the non-union in a surgical procedure (Verboket et al., 2018). Another ongoing clinical trial, Maxibone^1^, is studying the safety and efficacy of autologous cultured stem cells and calcium phosphate biomaterials in alveolar bone augmentation (Gjerde et al., 2018).

#### Megaprothesis

After trauma or resection of a malignant or benign aggressive tumor, the reconstruction of large bone defects is necessary to prevent amputation. The use of metal megaprotheses began in the 70s, and in the 90s, it became popular. Megaprotheses replace the affected bone tissue instead of regenerating bone tissue and there has been a significant evolution of their components since inception in order to ensure corrosion resistance, to avoid fractures of the material, for better fixation, and to guarantee osseointegration. Modular megaprostheses today allow the association of different components to customize large bone defect reconstruction (Hattori et al., 2011). Prostheses may have a coating of hydroxyapatite and silver for osseointegration and to prevent infection and various studies have shown excellent limb survival after surgery with a follow up of up at 20 years (Mittermayer et al., 2001; Gosheger et al., 2006; Jeys and Grimer, 2009; Shehadeh et al., 2010). There are two significant complications after reconstruction with megaprosthesis, mechanical and non-mechanical complications. Implant design may cause inherent mechanical complications and those reported in the literature include aseptic loosening, failure of soft tissue attachments, and prosthesis stem fractures. These complication rates are between 5 and 48%, as described in the literature (Ahlmann et al., 2006; Gosheger et al., 2006; Holl et al., 2012) and robust modular megaprostheses have helped to reduce this mechanical complication (Choong et al., 1996; Jawad and Brien, 2014). Non-mechanical complications include infection, tumor relapse, and wound healing disorders. Infection and wound necrosis are common complications in oncological cases due to malnutrition, immunosuppression, lack of local tissue vascularization, and extensive implant reconstruction (Jeys et al., 2005; Jeys and Grimer, 2009; Pala et al., 2015). Silver coated prosthesis, antibiotics therapy, and meticulous surgery techniques may reduce these complications; however, non-mechanical complications are the primary threat in large bone defect reconstruction using megaprosthesis.

### Masquelet Induced Membrane Technique

The induced membrane method known as the Masquelet technique consists of a two-stage operative procedure. The first stage includes a debridement of the defect site, soft-tissue repair and the insertion of a cement spacer composed of polymethyl methacrlyate (PMMA) that allows the maintenance of the bone height and stability, and the formation of a pseudosynovial membrane due to a foreign-body reaction. In the second step, performed 6–8 weeks later, the cement spacer is removed and the cavity is refilled with an autologous cancellous bone graft (e.g., from the iliac crests), while preserving the induced membrane. This membrane has various functions, in particular it prevents the resorption of the cancellous bone graft, supports vascularization and corticalization, and functions as a delivery system for osteomodulatory and angiogenic growth factors like transforming growth factor (TGFβ), bone morphogenetic protein 2 (BMP2) and vascular endothelial derived growth factor (VEGF) (Masquelet, 2003; Pelissier et al., 2004; Masquelet and Begue, 2010). This innovative technique is indicated in acute and chronic infected or non-infected massive bone defects of any size (4–25 cm) and shape, at different anatomical sites in children and adults (Masquelet et al., 2000; Azi et al., 2019). Its consolidation rate varies from 82 to 100% with delays ranging from 4 months to 1 year. The main complications include infection, failure of a step in the surgical procedure (persisting infection or non-union), re-fracture and severe bone graft resorption (Morelli et al., 2016; Han et al., 2017). Different studies reported the Masquelet’s approach as effective, for instance Sivakumar et al. (2016) and Mathieu et al. (2019) described the use of the induced membrane technique in the management of large bone defect reconstruction in open fractures of the femur, tibia, and fibula bones. A recently published review reported the application of the induced membrane technique in patients with osteomyelitis, suggesting this technique is an excellent alternative to solve long bone infected defects by controlling the local infection (Careri et al., 2019).

### Ilizarov Method

The Ilizarov method is a convenient tool for the treatment of patients suffering from poly−trauma conditions, with multiple fractures, osteomyelitis, and infected non-unions. The principle of the Ilizarov’s technique is to stimulate bone growth by bone distraction that produces neovascularization, and stimulates new bone formation (Aronson et al., 1989; Ilizarov, 1990). The surgical procedure consists of the use of an external circular fixator and a corticotomy. The external fixator stabilizes the bone and allows early weight-bearing. A distraction of 0.25 mm, four times per day, commencing after a delay of 5 to 10 days post-surgery is performed and an osteogenesis activity occurs in the bone gap (Spiegelberg et al., 2010). The length of bone that can be produced by this technique is up to 20 cm per limb segment. Barbarossa et al. conducted a study of 30 patients with osteomyelitis and infected non-union of the femur treated with the Ilirazov technique and reported efficacy in saving the limbs with osteomyelitis (Barbarossa et al., 2001). Large blood vessels expressing smooth muscle α-actin were shown to co-express BMP2 which was involved in enhancing osteogenic activity at the site (Matsubara et al., 2012). The Ilizarov’s bone distraction technique also offers the possibility of correcting a defect of axis, and allows a lengthening of the limb, however, it has associated drawbacks such as several weeks lag time required to heal large segmental defects, with extended hospital recovery and discomfort for patients, as well as risks of osteomyelitis along the transcutaneous pins.

## Future Directions in Large Bone Defect Reconstruction

### In-Patient Bioreactor

The principle of this approach is to use the patient as their own bioreactor, and entail the fabrication of a customized bone graft utilizing medical imaging and 3D printing, and the implantation of these osteoinductive materials in ectopic sites such as under the skin or in muscles. After several weeks, the pre-fabricated bone graft is used for large skeletal reconstruction. The possibility of producing substitute organs or body parts inside human bodies, therefore using the body as a living bioreactor was introduced (Cao et al., 1997; Vacanti and Langer, 1999) and Orringer et al. (1999) first treated an angle to angle mandible and total lower-lip reconstruction with a prefabricated osteocutaneous flap. A dacron-polyurethane tray was packed with autologous cancellous bone graft and with BMP7. This tray was implanted in the fascia above the scapula for generating a composite pre-fabricated flap (Orringer et al., 1999). Warnke et al. (2004) developed the bone-muscle-flap prefabrication technique for maxillofacial reconstruction. They grew a subtotal mandible composed of a titanium mesh cage filled with bone bovine mineral blocks, bone mineral granules associated with BMP7, and autologous bone marrow concentrated cells inside the latissimus muscle and vascularization was provided by the thoracodorsal pedicle. Seven weeks postoperatively, the prefabricated bone muscle flap was microsurgically transplanted with its vascular pedicle in the mandible. Vascular supply of the flap was successfully maintained. A favorable aesthetic and functional outcome was obtained (Warnke et al., 2004). Mesimaki et al. (2009) then described a 3 step surgery method to reconstruct a large bone maxillary defect by forming a prevascularized construct by filling a titanium mesh cage with autologous adipose-derived stem cells (ASCs), BMP2 and beta-tricalcium phosphate (β-TCP) granules and inserting it in the patient’s left rectus abdominis muscle, with vascularization provided by the inferior epigastric artery, and subsequent transplantation for maxillary bone reconstruction. Other studies described the use of the pectoralis major – hydroxyapatite blocks flap, pedicled using the thoracoacromial artery, for mandible reconstruction (Heliotis et al., 2006; Tatara et al., 2014). A further alternative comprised a polymethylmethacrylate chamber filled with autograft implanted against the periosteum of the iliac crest which was transplanted to the mandibular site after 8 weeks, with the donor periosteum sutured with the local periosteum to reestablish the vascularization (Cheng et al., 2006). Kokemueller et al. (2010) reported hemimandible reconstruction by utilizing cylinders of β-TCP loaded with cells and morcellized autologous bone graft that were implanted in the latissimus dorsi muscle with a central vascular bundle and transplanted after 6 months. The main advantage of the patient bioreactor method compared to the alternative surgical treatments proposed for large bone defects reconstructions (e.g., autologous vascularized fibula, iliac crest) is that it avoids the process of harvesting native bone and creating further skeletal defects. However, this method does not apply to emergency cases and requires at least two surgical sites.

### 3D Printing Techniques and Production of Personalized Surgical Guides and Scaffolds

**FIGURE 3:**
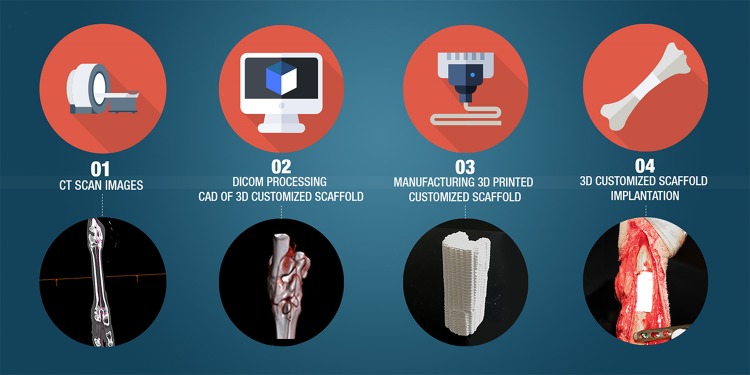
Workflow involved in customizable bone construct fabrication. (1) CT scans of the patient’s bone are acquired. (2) Computer aided software enables the processing of CT images in order to (3) 3D print personalized scaffolds for (4) bone defect reconstruction. The lower panel illustrates a real large bone defect reconstruction in a sheep metatarsal bone model.

3D printing is an emerging technology that permits the manufacture of complex-shaped structures with high precision using layer-by-layer printing of different materials. As illustrated in Figure 3, the structures of the defects to be reconstructed in patients are identified based on digital images obtained from a computed tomography (CT) scan or magnetic resonance imaging (MRI), and by using computer-aided design (CAD) software, 3D printing technology and bioprinting 3D medical models can be developed (Colin and Boire, 1997; Winder and Bibb, 2005). The 3D printing technologies used for polymer scaffold construction are: (1) fused deposition modeling (FDM), (2) selective laser sintering (SLS), and (3) stereolithography (SLA). The FDM method is the most popular technique developed in the 1980s and based on construction by melting deposition. The material commonly used is a thermoplastic polymer, in powder or filament format, which feeds an extruder tip that melts the plastic and at its exit is deposited on a surface at a much lower temperature so that it solidifies rapidly. The extruder tip moves in the *x* and *y* planes to print layer by layer the pattern of the scaffold (Xu et al., 2014). The resolution of the printed construct is defined by multiple factors: nozzle diameter, print speed, and number and height of the layers (Yang et al., 2018). This technique is simple, rapid, and cost-effective, however, there are limited choices of biocompatible, medical-grade thermoplastic polymers available. SLS uses a CO_2_ laser that sinters, layer by layer, the material in a powder state, forming the final piece. The final piece needs to be cleaned to withdraw the powder excess and to provide smoothness to the construct surface. SLS allows the fabrication of large and sophisticated structures (Deckard, 1989; Mazzoli, 2013). SLA produces 3D models by tracing a beam of UV light or a laser on a base of a photosensitive resin that polymerizes (Mondschein et al., 2017). The main benefit of this 3D printing technology is the high level of detail and the excellent surface resolution (Ji et al., 2018).

### 3D Printing in Bone Tissue Engineering Applications

3D printing prototype models can significantly assist with pre-operative evaluation and intraoperative procedures, for example for the use of surgical guides in mandibular reconstruction with osteocutaneous flaps (Bosc et al., 2017; Dupret-Bories et al., 2018). These studies showed the advantages of using 3D printed preoperative models and surgical guides including a reduction in operating time, flap ischemia, morbidity and associated complications such as infections. Many studies describe the use of 3D printing scaffolds for bone tissue engineering (Kao et al., 2015; Petrochenko et al., 2015; Saito et al., 2015; Wang et al., 2015). Various types of ceramics, like HA, β-TCP, alpha-tricalcium phosphate (α-TCP), BCP, bioactive glasses, and more, have been used in recent years for the development of 3D printed scaffolds (Vorndran et al., 2008; Suwanprateeb et al., 2009; Klammert et al., 2010b), however, these materials are often brittle and do not match the mechanical properties of bone. To obtain similar mechanical strength to bone, bioceramics can be blended with polymers, such as cellulose, poly(D,L-lactic acid-co-glycolic acid) or polycaprolactone (PCL), before being printed (Liao et al., 2011). PCL is a polymer, with FDA approval that is widely used in 3D printing. It has a low melting temperature (60°C) (Wang et al., 2015), favorable viscoelasticity, and is biodegradable. Its slow degradation and high stiffness make PCL one of the preferred polymers for the manufacture of a 3D printing scaffold for bone tissue engineering (Brunello et al., 2016). The use of CT to create anatomically accurate scaffolds of calcium phosphate for cranial defects and alpha-TCP for maxillofacial deformities reconstruction have been described (Saijo et al., 2009; Klammert et al., 2010a). Direct ink writing (DIW), also called robocasting, has been one of the most studied and commonly used techniques for the development of 3D bioceramic scaffolds. DIW is an extrusion-based additive manufacturing method, in which a liquid-phase ink containing a high volume content of ceramic powder is dispensed through a nozzle, following a digitally defined pattern to create a 3D construct in a layer-by-layer manner (Lewis, 2006; Feilden et al., 2016). The chief advantages of DIW is that it applies to a wide range of bioceramics and it is possible to control pore size, pore orientation, and lattice design of the printed scaffold. Moreover, it is a high speed, simple and economic technique (Michna et al., 2005; Miranda et al., 2006) and has been used to create a hydroxyapatite scaffold for possible use in maxillofacial reconstruction (Cesarano Iii et al., 2005).

The main advantage of 3D printing is direct control over both the microarchitecture and complex anatomical structure. These 3D printed models allow the manufacture of customized scaffolds that mimics the patient’s anatomy (Wubneh et al., 2018). However, there are different challenges in the translation of 3D printing bioceramics to clinical application. Firstly, 3D printed bioceramics are brittle and not suitable for load-bearing clinical applications. Secondly, the fabrication of a large-size scaffold for large bone defect reconstruction is time-consuming and expensive. Moreover, for producing these 3D printed bioceramics, toxic solvents, and high-temperatures are used in the printing procedures which may compromise cell viability (Rodríguez-Lorenzo et al., 2001; Lewis et al., 2006; Trombetta et al., 2017; Wen et al., 2017; Chen et al., 2019). There have been multiple *in vivo* animal studies conducted with 3D printed customized scaffolds for bone regeneration (Park et al., 2018; Choi et al., 2019), however, these techniques are still in a developmental stage for clinical application and not capable of fabricating large-sized bioceramic scaffolds.

### 3D Bioprinting a Custom Living and Vascularized Bone Graft

Bioprinting is another 3D printing technique that uses cell-laden hydrogels to print structures that after a period of maturation, will develop complex tissues, such as skin, cartilage, and bone. Vascularization can be aided by the incorporation of angiogenic growing factors or endothelial cells into bio-inks (Kolesky et al., 2014; Fahimipour et al., 2017; Benning et al., 2018). Three major procedures are the most used in bioprinting: inkjet, extrusion, and laser-assisted bioprinting. For tissue engineering applications, thermal and piezoelectric inkjet bioprinters are commonly used. In the piezoelectric inkjet bioprinter system, a piezoelectric crystal is used to create different potentials which generates pressure that allows the bioink ejection in the form of droplets. In thermal inkjet bioprinting, the printhead is heated up to 300°C that generates small air bubbles that produce pressure pulses to eject bioink droplets. The size of droplets depends on multiple factors, such as ink viscosity, the frequency of the current pulse and the gradient of the temperature (Hock et al., 1996; Hudson et al., 2000; Cui et al., 2012). The significant advantage of inkjet bioprinting is its rapid fabrication (Murphy and Atala, 2014). In extrusion bioprinting, a bioink is dispensed using pneumatic air pressure or mechanical systems composed of a screw or a piston. The flow of the bioink is more controlled in the mechanical system due to the action of the screw. With the pneumatic air, an interrupted filament is ejected, allowing high precision in the printed construct. Cells are exposed to high mechanical stress during this procedure, which may affect cell viability (Mandrycky et al., 2016). Extrusion bioprinting allows printing of different types of inks with different viscosities (Ozbolat and Hospodiuk, 2016; Paxton et al., 2017). The main disadvantage of this technique is that the high viscosity of the bioink or cell aggregation can clog the printer tip. Laser bioprinting consists of the interaction of a pulsed laser source with a ribbon. This ribbon contains an energy-absorbing layer, and below it, the bioink is located. A collector-slide receives the droplets of hydrogel created by the dynamic jet facilitated by the energy deposition that is created by the laser effect in the ribbon. In this procedure cells are not submitted to a mechanical stress (Gruene et al., 2011; Unger et al., 2011) and it is a nozzle-free cell printing technique with high resolution. Although 3D bioprinting brings the potential of producing a customized and vacsularized living bone transplant, this biofabrication technique has not yet been tested in clinical cases. Numerous remaining challenges such as obtaining optimal cell numbers, adequate cell viability and spatial cell differentiation of the 3D construct, as well as reconnection to the local vasculature are yet to be resolved.

## Conclusion

In this review, the current bone reconstructive options for large skeletal defects such as autologous, allogeneic, biological and synthetic bone grafts are presented, as well as the future directions in bone tissue engineering that take advantage of 3D printing. The current gold standard technique for large bone defect reconstruction is autologous free vascularized bone flap transplantation that contains the patient’s cells, growth factors, and a vascularization bed. However, its main disadvantages are donor site morbidity, laborious microsurgery, and the need to sculpt the construct to the anatomy of the bone defect. Alternatively, allogeneic bone is also used to reconstruct large bone defects, but it is less osteogenic than autologous bone and may induce immunogenic rejection and transfer of disease. 3D printing technologies permit the fabrication of personalized bone grafts and the improvements in the incorporation of cells, growth factors, and vasculature may revolutionize bone tissue regeneration.

## Author Contributions

LV, CK, and MB wrote the main manuscript text and prepared the figures. AH edited the manuscript. PL edited the manuscript and prepared the figures. All authors have read and approved the final version of the manuscript.

## Conflict of Interest

The authors declare that the research was conducted in the absence of any commercial or financial relationships that could be construed as a potential conflict of interest.

## Footnotes

1 www.maxibone.eu
